# Nanoparticles mediated tumor microenvironment modulation: current advances and applications

**DOI:** 10.1186/s12951-022-01476-9

**Published:** 2022-06-14

**Authors:** Ganji Seeta Rama Raju, Eluri Pavitra, Ganji Lakshmi Varaprasad, Sai Samyuktha Bandaru, Ganji Purnachandra Nagaraju, Batoul Farran, Yun Suk Huh, Young-Kyu Han

**Affiliations:** 1 Department of Energy and Materials Engineering, Dongguk University–Seoul, Seoul, 04620 Republic of Korea; 2 Department of Biological Engineering, Biohybrid Systems Research Center (BSRC), Inha University, Incheon, 22212 Republic of Korea; 3 Baton Rouge General Hospital, Baton Rouge, LA 70809 USA; 4 School of Medicine, Division of Hematology and Oncology, University of Alabama, Birmingham, AL 35233 USA; 5 Department of Hematology and Medical Oncology, Winship Cancer Institute, Emory University, Atlanta, GA 30322 USA

**Keywords:** Nanoparticles, Tumor microenvironment, Immunotherapy, Chemotherapy, Drug delivery, Photodynamic therapy

## Abstract

The tumor microenvironment (TME) plays a key role in cancer development and emergence of drug resistance. TME modulation has recently garnered attention as a potential approach for reprogramming the TME and resensitizing resistant neoplastic niches to existing cancer therapies such as immunotherapy or chemotherapy. Nano-based solutions have important advantages over traditional platform and can be specifically targeted and delivered to desired sites. This review explores novel nano-based approaches aimed at targeting and reprogramming aberrant TME components such as macrophages, fibroblasts, tumor vasculature, hypoxia and ROS pathways. We also discuss how nanoplatforms can be combined with existing anti-tumor regimens such as radiotherapy, immunotherapy, phototherapy or chemotherapy to enhance clinical outcomes in solid tumors.

## Introduction

The tumor microenvironment (TME) of solid tumors remains one of the main barriers to successful therapeutic intervention in cancer treatment. The TME is a heterogeneous mass composed of various cell subtypes, namely immune cells, endothelial and inflammatory cells, fibroblasts and lymphocytes surrounded by the extracellular matrix (ECM), stroma and vasculature, as well as chemokines, organelles and secreted proteins [1–3]. The TME is implicated in tumor initiation, metastasis and recurrence. Immune cells are responsible for innate versus adaptive immunity. Innate immunity is mediated by cells such as macrophages and dendritic cells (DCs), which exert both anti- and pro-tumorigenic responses based on signaling pathways and chemokines in the TME. Adaptive immunity can specifically target tumor cells and is more effective in eradicating tumors [2].

Fibroblasts are predominant components of the TME and contribute to all cancer stages. Activated fibroblasts are called cancer-associated-fibroblasts (CAFs) [3]. They shape the TME by building large portions of the (ECM) and enhance tumorigenesis and angiogenesis through cytokines, growth factors and matrix metalloproteinases, which induce TME remodeling [2, 3]. CAFs silence cytotoxic T cells and recruit inflammatory lymphocytes that promote tumor progression [4]. CAFs can also reconvert anti-tumorigenic milieus into pro-metastatic environments via stroma derived factors. For instance, fibrinogen-like protein 2 can enhance pro-tumorigenic CAF activity, which increases myeloid-derived suppressor cells (MDSCs) activity through CXCL12, thus enhancing cancer progression [5]. Because of these various abnormal signaling cascades, the tumorigenic TME is characterized by an aberrant vasculature that promotes hypoxia. Hypoxic states can modify TME activity, stimulate tumorigenesis and limit therapeutic effects [2]. Since the TME modulates drug function and penetration, it can aggravate drug resistance by protecting pro-proliferation factors in the TME and tumor mass [2]. Hence, the complex matrix of interactions between cancer cells and TME components constantly reshapes tumor development, therapeutic efficiency, and potential drug resistance mechanisms. In view of these profound influences, targeting aberrant TME components has emerged as an attractive strategy for treating solid tumors, particularly aggressive and recalcitrant tumors such as gastrointestinal (GI) cancers.

Immune modulating regimens include immunotherapeutic approaches like pembrolizumab, which targets programmed cell death protein-1 but elicits limited effects in GI cancers such as colorectal or pancreatic cancer (CRC and PC) due to the obstructive TME [6, 7]. Other strategies include chimeric antigen receptor T (CAR-T) cell therapy, which has restricted responses in lung cancer and melanoma [2]. The failure of these approaches in GI cancers has prompted researchers to develop novel strategies aimed at potentiating the effects of solid and targeted therapies [8]. Nanoparticles (NPs) have garnered attention as a potential tool for enhancing immune therapies due to their prolonged retention time and targeted delivery, which reduces cytotoxicity [9]. NPs are versatile agents that can target major TME components to modulate the immunosuppressive environment. For example, angiogenesis and rapid tumor growth result in hypoxia and immunosuppression by activating T_regs_ and MDSCs, which secrete vascular endothelial growth factor (VEGF) and transforming growth factor (TGF)-β [2]. These factors suppress DC function and stimulate pro-tumorigenic M_2_ macrophage phenotypes, resulting in aberrant fibrosis [2]. Specialized NPs can specifically target these TME components to reprogram the immunosuppressive TME into an immunosupportive environment and resensitize cells to immunotherapy. NPs can be used as passive drug delivery platforms with prolonged drug retention time. They can accumulate in neoplastic tissue versus normal cells due to increased enhanced permeability and retention (EPR) effects, aberrant lymphatic drainage and leaky vasculature. Furthermore, they can be actively targeted to tissues by modifying their structure and conjugating them to specific ligands, thus increasing their versatility, compatibility and efficiency [10, 11]. Hence, nanoplatforms of varying chemical composition, shapes and sizes can be tailored and used as effective combination strategies to deliver targeted therapies to specific regions within the TME. Nanodrugs such as Abraxane and Doxil have already been approved by the Food and Drug Administration (FDA) for clinical use [12]. In this review, we discuss recent advances in the field of cancer nanotherapeutics, focusing on nanoscale platforms designed to target various TME components, such as tumor-associated macrophage (TAM) reprogramming, ECM and tumor vasculature remodeling and hypoxia relief. We also explore how these innovative solutions can be used to improve the potency and efficiency of current treatment regimens such as immunotherapy and chemotherapy.

## Targeting the tumor immunosuppressive components

**Fig. 1:**
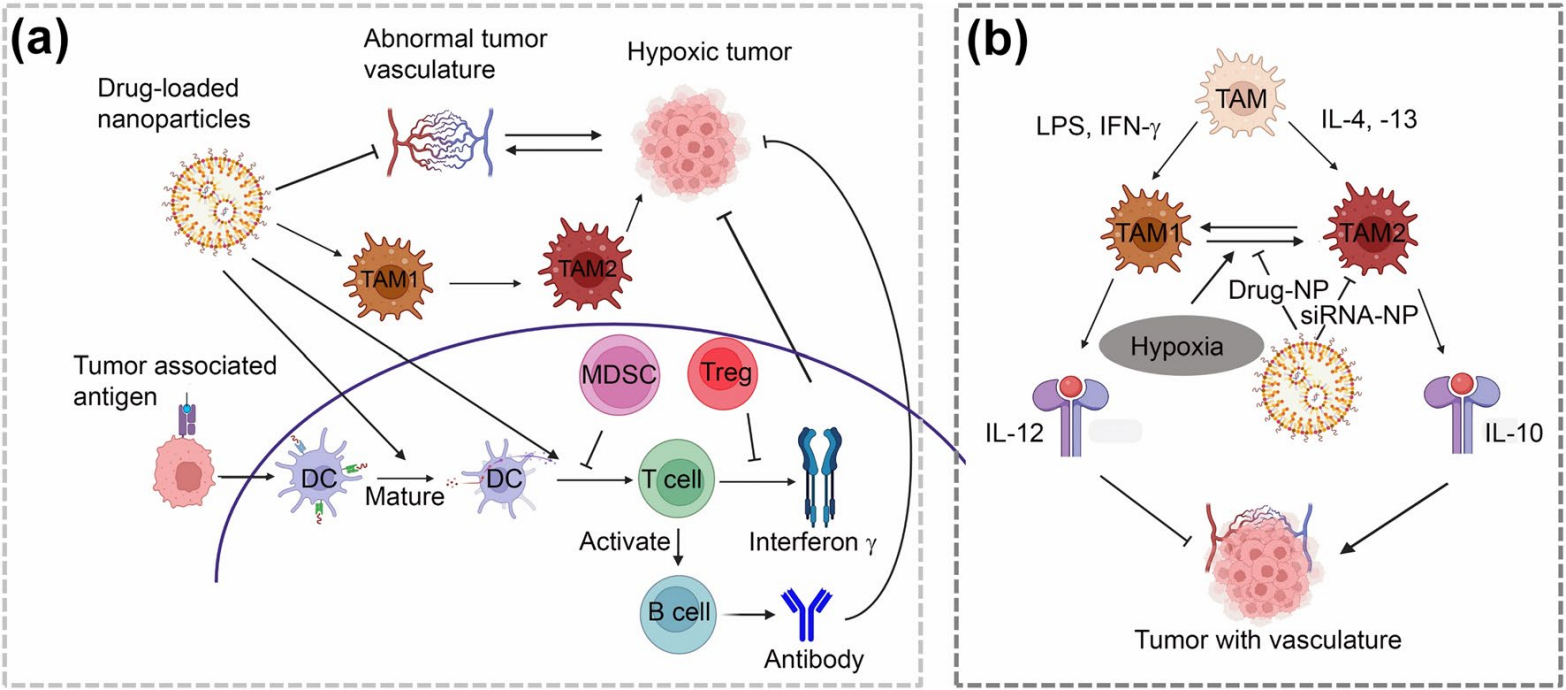
**a** Drug loaded NPs can inhibit tumor growth through multiple pathways: Drug loaded NPs can inhibit hypoxic tumor growth by targeting and reprogramming the abnormal tumor vasculature. Additionally, they can reprogram M_2_ macrophages into M_1_ phenotypes, which reactivate the immune response and inhibit tumor growth. Drug loaded NPs can also activate mature DCs, leading to the activation of B cells through T cells and the production of antibodies that can target and eradicate tumor cells. These NP-stimulated pathways can thus overcome the inhibitory effects exerted by MDSCs on mature DCs and the inhibitory effects of Tregs on INF-γ, thus killing tumor cells. **b** TAMs regulate the tumor vasculature: TAMs inhibit tumor vasculature through M_1_ and IL12. Hypoxia induces TAM_1_ to TAM_2_ conversion, thus promoting tumor vasculature through IL-10. Drug-loaded NPs can inhibit the conversion of TAM_1_ to TAM_2_ and reduce tumor growth. SiRNA-loaded NPs can also suppress TAM_2_ activity

The combination of immune-oncology and nanomedicine has been evaluated in various pre-clinical and clinical trials. Sun et al. [12] have proposed a model termed the “cancer-immunity” cycle to illustrate immune-targeting approaches based on nano-immunotherapy. The cancer-immunity cycle consists of four steps and begins with antigen secretion from tumor cells. Antigens are then processed by antigen-presenting cells (APCs) and displayed to naïve T cells to generate cytotoxic T cells, which migrate to identify and eradicate tumor cells using toxic molecules like granzyme B or perforin. This initial cascade triggers the release of more antigens, initiating a second tumor eradication cascade [12]. Cancer nanodrugs have been exploited to enhance the effects of immunotherapy. For instance, they have been used to induce antigen release from tumor cells through loading compounds such as chemotherapeutic or photothermal agents that elicit immunogenic cell death (ICD) into nanocarriers [13]. The feasibility of this strategy was demonstrated using oxaliplatin-loaded nanocarriers in a PC mouse model. The study revealed that oxaliplatin-loaded NPs enhanced apoptosis compared to the free drug and improved immune-activation by increasing tumor T cell infiltration, DC maturation and interferon gamma (INF-γ) secretion, thus demonstrating the therapeutic efficacy of oxaliplatin NPs [14]. Furthermore, NPs incorporating both tumor antigens and/or adjuvants have been developed as cancer vaccines that mount immune responses targeting self- and neo- antigens arising from somatic mutations within the cancerous mass [15]. NPs based on cancer cell-derived membranes and fused with erythrocyte cell membranes have also been tailored and found to elicit antigen responses in mouse models [16]. Moreover, NP-tailored solutions have been devised to improve the last step in the cancer immunity cascade, namely the recognition and eradication of tumor cells by cytotoxic T lymphocytes. For instance, liposomes loaded with IL-15 and 21 were synthesized using maleimide groups. The liposomes successfully attached to T cells surfaces using thiol groups and

slowly secreted cytokines to activate T cells. T cells treated with these liposomes exhibited increased endurance compared to non-treated cells, resulting in improved therapeutic responses [17]. As these various studies show, NP-based platforms can be successfully incorporated into immunotherapy regimens to ameliorate the delivery and efficiency of specific drugs. The schematic (Fig. 1a) illustrates the multiple pathways for inhibiting the tumor growth by drug loaded NPs. However, those strategies require stringent testing in clinical settings to establish their true efficacy in human patients.

## Nanoparticle strategies for immune modulation

### Using nanoparticles to target TAMs

Macrophages play key roles in wound healing and regeneration, homeostasis and immunity. As differentiated cells within the phagocyte system, they mediate phagocytosis and engulf foreign substances not recognizable as normal cells [2, 18]. Following signal exposure, macrophages differentially transform into M_1_ or M_2_ subtypes. Exposure to LPS and IFN-γ polarizes macrophages into classical M_1_ macrophages that secrete interleukin (IL)-12, which inhibits cancer progression (Fig. 1b). Macrophages exposed to IL-4 and 13 transform into alternative M_2_ macrophages, which secrete IL-10 and promote tissue repair and healing, therefore facilitating tumor growth [2]. Because of these two alternative phenotypes, macrophages are viewed as “double-edged swords” displaying both tumor enhancing and inhibitory effects depending on immune context. TAMs stimulate anti-cancer immunity during tumor initiation stages but become hubs of immune suppression and angiogenesis during advanced cancer stages [19]. This behavioral discrepancy is due to the plastic nature of macrophages. TME alterations during tumor growth cause TAMs to undergo a transition from M_1_ to M_2_-like phenotypes. Reprogramming TAM polarization could thus influence TAM function and improve their anti-tumor effects [2]. Nano-based solutions specifically targeting TAMs could thus regulate their polarization and might represent a promising cancer immunotherapy strategy if successfully translated to the clinic.

**Fig. 2:**
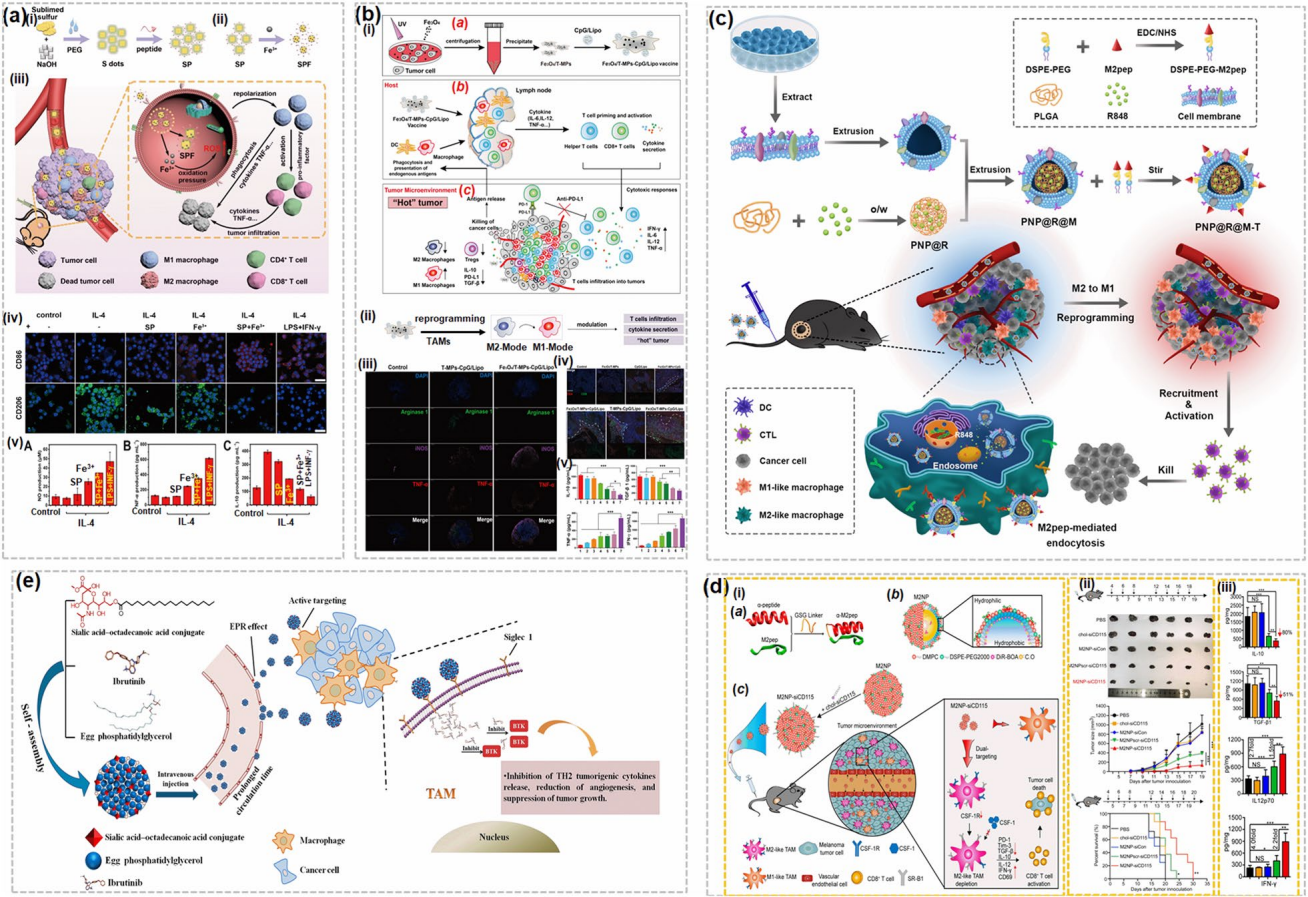
Reprogramming of TAM polarization using nanodrug delivery systems: **a** Iron nanotraps reprogrammed macrophages: (i and ii) schematic representation of the development of iron nanoptraps with the conjugation of TAMs targeted peptides on the S dots and simultaneous absorption of iron, (iii) corresponding mechanism for the reprogramming of TAMs, and (iv) confocal microscope images of the M2 and M1 macrophage markers and (v) estimation of the generation of nitric oxide (NO), TNF-α and IL-10. Copyright 2021 by Sang [21]. **b** Fe_3_O_4_/T-MPs-CPG/Lipo reprogrammed TME: (i) (*a*) schematic for the development of CpG loaded liposomes conjugated tumor-derived antigens decorated nano-Fe_3_O_4_ microparticles, (*b*, *c*) stimulation of APC (DC and macrophage) progression, T cells activation, production of pro-inflammatory cytokine and boosting of antitumor response to immunotherapy by reprogramming the TME (corresponding TME modulation in the form of schematic is presented in Fig. (ii)). Immunofluorescence images of B16F10 tumor section (iii) after treatment with and without Fe_3_O_4_ nanoparticles, and (iv) after injection with various formualtions. (v) Enzyme-linked immunosorbent assays of IL-10, TGF-β1, TNF-α, and INF-γ. Copyright 2019 by Zhao [22]. **c** Schematic representation of the development of PNP@R@M-T and their reprogramming of TMEs by selective adaptation of M2-like macrophages. Copyright 2021 by Zhang [23]. **d** Molecular targeted immunotherapy for M2-like TAMs: (i) schematics for the (*a*) development of α-M2pep, (*b*) components of M2-like TAMs targetingcore-shell fluorescent lipid nanoparticle, and (*c*) in vivo immune regulation mechanism, (ii) inhibition effect on tumor growth, and (iii) Enzyme-linked immunosorbent assays of IL-10, TGF-β1, IL-12p70, and INF-γ Copyright 2017 by Qian [24]. **e** schematic representation of the tyrosine kinase in TAMs using the nanocomplex SA/IBR/EPG. Copyright 2019 by Qiu [25]

Various nano-based strategies aimed at regulating TAM polarization have been developed and tested (Fig. 1b). For example, Zanganeh et al. [20] discovered that off-label use of iron oxide NPs such as ferumoxytol, currently approved for iron deficiency treatment, can elicit a transition from M_2_ to pro-inflammatory M_1_ phenotypes in solid tumor tissues, thus suppressing tumor growth. This suggests that off-label ferumoxytol could enhance macrophage-regulating immunotherapies [20]. Another iron-based nanostrategy consisted in developing nanotraps by altering TAMs-targeted proteins on S dots (Fig. 2a(i and ii)) [21]. The nanotraps harbored many oxygen-containing groups on their surfaces, thus allowing them to adsorb, capture and specifically deliver iron to TAMs (Fig. 2a(iii)). When internalized within lysosomes, the nanotraps successfully released iron and induced oxidative pressure, thus shifting TAMs from M_2_ to M_1_ subtypes (Fig. 2b(iii)). Activated M_1_ secreted H_2_O_2_, further facilitating iron release for their repolarization and re-education. Additionally, nanotraps of ultra-small sizes achieved deeper tumor penetration, more efficient TAM polarization and improved immune responses [21]. The expression of M_2_ (CD206) and M_1_ (CD86) macrophage markers (Fig. 2b(iv)) was observed to enhance the generation of nitric oxide, TNF-α and IL-10 (Fig. 2b(v)). This study thus shows that endogenous substances can be exploited to enhance immunotherapy. Advanced nano-based approaches have also been used to develop an effective anti-tumor vaccine to reprogram the TME. Tumor-derived antigenic microparticles (T-MPs) carry cytosolic material and share markers and biological characteristics with their parental cancer cells. Their inner cavities can transport and deliver specific cargo to parental neoplastic nests through self-recognition [22]. Additionally, T-MPs retain highly immunogenic cancer-specific antigens and expose free.

sulfhydryls that can be chemically altered [22]. Consequently, T-MPs represent promising and versatile immunogenicity carriers. Zhao et al. [22] devised a potent cancer vaccine using a T-MP strategy that can exert anti-tumor effects in various solid tumor models. The related schematic is shown in Fig. 2b(i). Exploiting T-MPs functionality, they loaded nano-Fe_3_O_4_ in T-MPs and tethered dense CpG/Lipo unto Fe_3_O_4_ /T-MPs surfaces to develop an anti-tumor vaccine incorporating tumor antigen, immunostimulant adjuvant and immunomodulator in a single vesicle (Fig. 2b(i*a*)). The designed vaccine induced APC maturation, increased cytotoxic CD8 + T lymphocyte and CD4 + T helper populations (Fig. 2b(i*b*)) and induced the release of immunostimulatory cytokines including tumor necrosis factor (TNF)-α and IL-12 both in cell and animal models (Fig. 2b(i*b* and *c*) & Fig. 2b(iii–v)). Importantly, the vaccine accumulated at tumor sites and reprogrammed TAMs into a tumor inhibitory M_1_ state, thus transforming a “cold” tumor into a “hot” phenotype (Fig. 2b(i*b* and *c*) & b(ii)). This interesting approach warrants testing in clinical settings to evaluate its anti-tumor efficiency [22]. He et al. [26] resorted to dual targeting strategies to overcome cancer-related immunosuppression. Their dual system consisted of MCMC plus hyaluronan (HA), which can target mannose and CD44 receptors on macrophages, decorated on surfaces of nano-delivery platforms. The dual targeting NPs displayed higher immunostimulatory activity compared to mono-targeting platforms, stimulated pro-inflammatory cytokines and successfully shifted macrophages to M_1_ phenotypes. The dual-targeting NP did not influence cancer cell growth however [26], indicating that this strategy requires additional fine-tuning. To ameliorate anti-tumor efficiency, Deng et al. [27] engineered NPs cloaked with natural killer cellular membranes, capable of enhancing anti-cancer responses and M_1_ polarization. NK-NPs successfully targeted tumors and induced M_1_ phenotypes, eradicated primary neoplasms and suppressed growth of distal tumors, indicating that NK-NPs could represent versatile and efficient immunotherapeutic strategies. Wu et al. [28] investigated the possible association between TAMP polarization and the sizes of iron-based agents and nanomaterials to refine the design of anti-cancer drugs. They used FeOOH nanorods of varying sizes coated with PAA to develop pFEOOH NRs. They found that pFEOOH NRs of shorter length exerted increased reprogramming abilities due to improved uptake into TAMs and higher iron dissolution. The short pFEOOH doxorubicin (DOX)-loaded NRs were used for anti-tumor treatment, achieving immunostimulant TME effects, ameliorated recruitment of immune cells and ICD, and tumor recurrence suppression. These results suggest that proper fine-tuning of nano-cargos and elucidation of their property/size-activity relationships can improve their therapeutic efficiency.

Zhang et al. [23] developed a biomimetic anticancer formula aimed at reprogramming M_2_ macrophages and enhancing antitumor immunity. To reprogram M_2_ subtypes, the group coated resiquimod (R848), a Toll-like receptor 7/8 agonist and powerful driver of macrophage polarization and reprogramming, with Poly (lactic-co-glycolic acid) (PLGA; renamed it as PNP@R) and introduced a B16-OVA membrane to encapsulate the PNP@R surfaces (PNP@R@M), as shown in Fig. 2c. This particular membrane expresses high levels of CD47, which protects the NPs from clearance by the RES. Furthermore, they modified the membrane using synthetic PEG, which reduces the interactions between blood proteins and NPs, thus shielding them from phagocytosis and aggregation. The final modified M_2_-like biomimetic macrophage-targeting NP (PNP@R@M-T) successfully reprogrammed M_2_ phenotypes in a selective fashion, resulting in enhanced lymphocyte infiltration and stimulation of host immunity [23]. Hence, this engineering strategy, which utilizes the therapeutic potential of Toll like receptors targeting through an improved formulation, might provide a promising approach for selectively and efficiently repolarizing and re-educating M_2_ macrophages. Researchers have also explored alternative strategies such as bacterial therapy to improve macrophage reprogramming. For example, Wei et al. [29] resorted to DOX-elicited immunogenic cell death (ICD) to enhance TAM polarizing immunotherapies. For this purpose, R848 and DOX were separately decorated with PLGA to tailor R848-containing PLGA NPs (PR848) and DOX-carrying PLGA NPs (PDOX). Glycol chitosan was utilized to link E. coli MG1655 and PR848 to generate PR848 NP-loaded E. coli (Ec-PR848). The resulting Ec-PR848 successfully accumulated at neoplastic sites through E. coli-elicited hypoxia targeting and repolarized M_2_ subtypes into tumor-inhibitory M_1_ phenotypes. Combination of Ec-PR848 and PDOX triggered ICD and enhanced M_2_ repolarization, thus improving T lymphocyte infiltration into cancerous pockets and suppressing tumor development [29]. Hence, this nano-based bacterial immunotherapy strategy could represent an innovative and promising solution for reprogramming TAMs and enhancing anti-tumor immunity. Another nano-based combination approach consisted of developing a versatile NP encapsulating two pro-drugs: a modified form of GEM and a STAT3 inhibitor (HJC0152). The prodrugs can release their GEM cargo intracellularly and respond to hypoxic milieus to yield HJC0152. STAT3 inhibition can reeducate the TME by reversing M_2_-TAM phenotypes, recruiting CTLs and downregulating Tregs. Additionally, the lipid alteration in GEM can relieve drug resistance to GEM. Hence, this versatile platform can exert synergistic therapeutic effects and warrants further investigation. As these various studies and nano-engineering achievements illustrate, nano-based immunotherapeutic approaches aimed at repolarizing TAMs are a viable strategy against solid tumor models. Translational studies will establish whether these technologies can successfully improve clinical outcomes in human cancer patients.

In parallel with TAM repolarization, other TAM-targeting strategies include inhibition of TAM function and survival. To test this strategy, Qian et al. [24] devised a nanocarrier exhibiting both M_2_-like and TAM targeting capacity (Fig. 2d). The dual-targeting M_2_ NP was designed to deliver siRNA M_2_-like TAMs (Fig. 2d(i)). Its function was regulated by an α-peptide, namely a scavenger receptor B type 1 (SRB1) targeting protein interlinked with the M_2_ binding peptide, M_2_Pep (Fig. 2d(i*a* and i*b*)). The two units (α-peptide and the M_2_Pep) exerted synergistic effects in the α-M_2_Pep fusion. The M_2_NPs demonstrated high affinity and specificity to M_2_-like TAMs compared to other macrophages and resulted in effective eradication of M_2_-like TAMs, subsequent decrease in tumor size and improved survival (Fig. 2d(i*c*-iii)). This dual-targeting approach also reduced production of immuno-inhibitory IL-10 and TGF-β, increased TNF-α and IL-12 expression (Fig. 2d(iii)), improved CD8 + T cell infiltration and restored T-cell based immune activity [24]. This study thus highlights the promising potential of nano-based dual targeting immunotherapies for clinical application. Qiu et al. [25] also used a similar approach to improve Ibrutinib (IBR) uptake by TAMs (Fig. 2e). IBR is a small molecule inhibitor of bruton tyrosine kinase (BTK), which is overexpressed in TAMS and contributes to immunosuppression and tumor growth. The group designed IBR nanocomplexes to prolong its circulation time and optimize its delivery. They further tailored IBR-loaded NPs decorated with stearic acid conjugates to efficiently deliver IBR into TAMs. The synthetic SA/IBR/EPG selectively delivered IBR to TAMs and blocked BTK activation, thus inhibiting Th_2_ tumor-promoting cytokine release and reducing tumor volume and angiogenesis [25]. As these findings suggest, modified small-molecule NP-based solutions might reduce clearance and improve the circulation time and uptake of small-molecule inhibitors by specific targets in the TME, thus enhancing immunotherapeutic outcomes. Hence, NPs offer valuable engineering opportunities that overcome the traditional obstacles of TAM-targeting immunotherapies, such as clearance and poor circulation, defective solubility and non-specific delivery. Furthermore, these nanosolutions might enhance the therapeutic efficacy of chemotherapies, radiation and immune therapies. However, given the complex nature of TAMs and their varied subtypes that can readily change based on TME context, these NPs might have restricted therapeutic effects despite their advanced bioengineering properties and should be tested in the clinic to evaluate their actual potency and efficacy against human cancers.

### Nanoparticle strategies for targeting cancer-associated-fibroblasts

The tumor stroma is composed of fibroblasts involved in tumor development through reciprocal crosstalk with tumor cells [11]. Fibroblasts modulate the arrangement and activity of healthy tissue by depositing the ECM and promoting tissue repair. They remain activated in cancer tissue and are known as CAFs [2]. CAFs hamper efficient drug delivery and promote drug resistance by upregulating the levels of α-smooth muscle actin (α-SMA), VEGF, and pro-angiogenic signals [2, 11]. Hence, targeting CAFs is an important strategy to remodel the TME, improve drug intake and overcome evasion mechanisms. Recent research has enabled the development of two types of CAF-based nanotherapeutic strategies: (1) CAFs disruption, which emphasizes the destruction of the barriers created by CAFs and (2) CAFs targeting, which uses CAFs to enhance therapeutic efficacy against cancer [11].

**Fig. 3:**
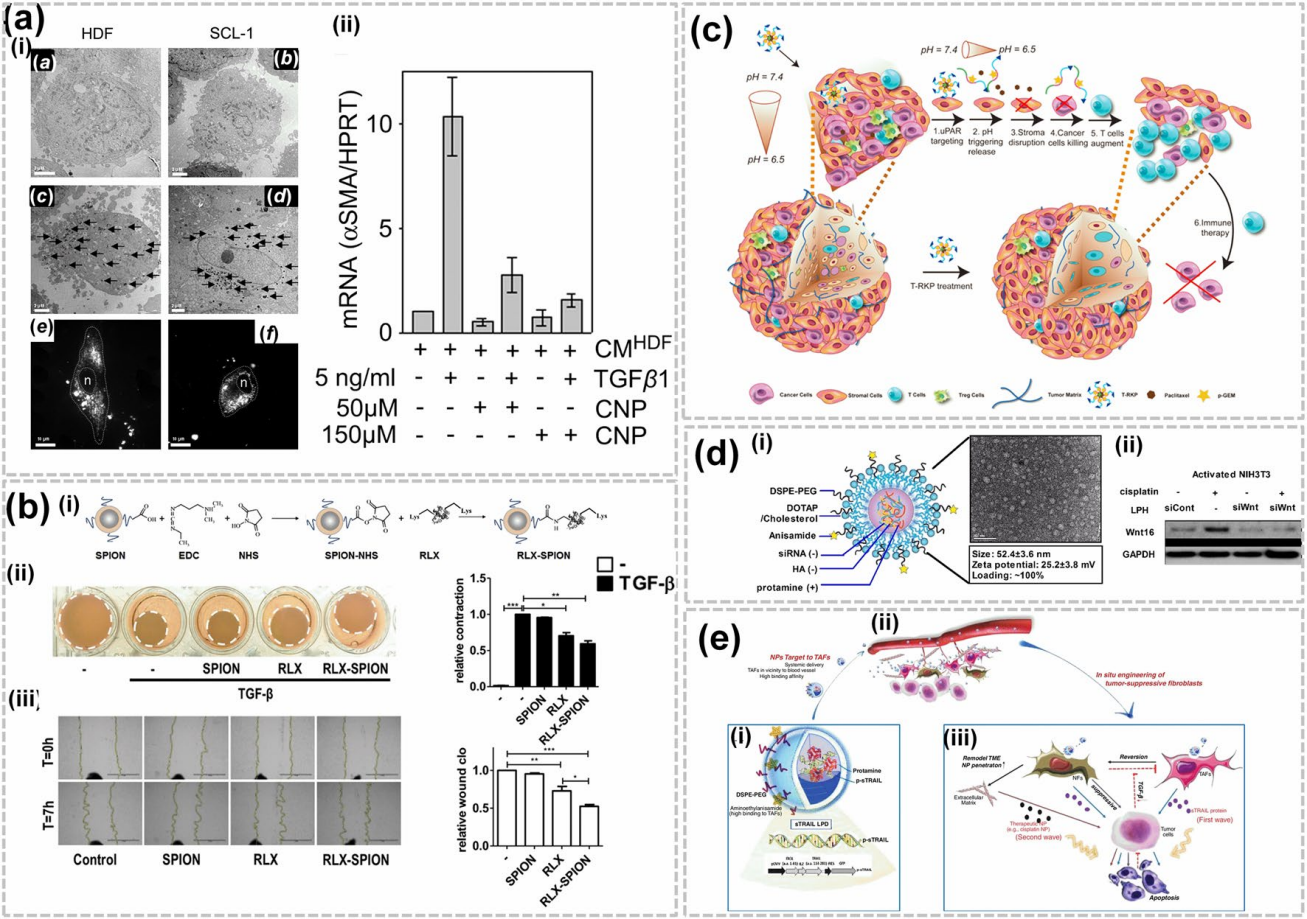
Destruction/targeting of CAFs: **a** (i) CeO_2_ morphology and corresponding fluorescent micrographs (*a–d*) cellular uptake of nanoceria, and (*e–f*) FITC-labelled nanoceria in human dermal fibroblasts (*e*) and sqauamous tumor cells (*f*), and (ii) TGFβ1-mediated mRNA expression of αSMA confirmed the downregulation after treated with nanoceria. Copyright 2011 by Alili [31]. **b** (i) SPION conjugated with relaxin-2 (RLX), (ii) photographs shows the effect of different concentrations of RLX on human PSCs when activating with 5 ng TGF-β relative contraction quantification is presented in bar diagram) and corresponding migration of human PSCs and relative wound healings are presented in Fig. (iii). Copyright 2018 by Mardhian [32]. **c** Schematic demonstrates the strategy for targeting the central part of TME and selectively disrupted α-SMA expression in the tumor core, destabilizing the internal core and eradicating tumor cells without affecting the outer stroma. Copyright 2019 by Chen [34]. **d** Gene transfection of siWnt NPs in in vitro studies. Copyright 2018 by Miao [35]. **e** Schematic illustrates the involved mechanism in targeting TAFs for ablating the tumor and reshaping the TME by reprogramming the CAFs. Copyright 2017 by Huang [36]

One of the strategies aimed at regulating CAFs consists in targeting myofibroblasts, which are modulated fibroblasts characterized by collagen contraction, secretion of pro-invasive signals and promotion of tumor evasion and survival [2]. Myofibroblast formation is driven by TGF-β1, which enhances reactive oxygen species (ROS)-induced α-SMA expression, and is suppressed by antioxidants [30]. Alili et al. [31] have shown that nanoceria can influence myofibroblast formation, cellular toxicity and tumor invasiveness (Fig. 3a). They demonstrated that nanoceria can downregulate α-SMA myofibroblastic cells (Fig. 3a(ii)) and inhibit the invasiveness of squamous cancer cells. The nanoceria specifically targeted tumor cells [31], indicating that they represent a safe and effective therapeutic strategy. Another myofibroblast-disrupting strategy consisted in targeting pancreatic stellate cells (PSCs), the main CAF precursors in the stroma. Mardhian et al. [32] devised superparamagnetic iron oxide NPs modified using relaxin-2 (Fig. 3b(i)), which suppresses TGF-β dependent differentiation of PSCs by silencing the pSmad2 cascade (Fig. 3b(ii)). The relaxin-2 NP enhanced targeted drug delivery, suppressed collagen deposition and retarded tumor growth (Fig. 3b(ii–iii)), indicating that nano-based strategies might improve drug pharmacokinetics and enhance stroma modulation. Several studies have also investigated nano-approaches aimed at eradicating stromal barriers and disrupting CAFs using drugs such as losartan or telmisartan [11]. For instance, Hu et al. [33] developed an injectable hydrogel NP solution loaded with losartan that improved drug encapsulation and allowed self-arrangement into elongated filaments harboring hydrophobic centers. The hydrogel was successfully retained in the cancer vicinity for up to 9 days, suppressing both collagen and CAFs production in orthotopic models. Additionally, losartan hydrogels amplified the therapeutic efficacy of Pegylated and DOX-loaded liposomes. This study indicates that losartan hydrogels can enhance pre-operative chemotherapy by modulating the stroma and warrants evaluation in other solid tumors. Chen et al. [34] developed a codelivery nanosystem that targets the deep pancreatic stroma while delivering nano-based chemotherapeutics (Fig. 3c). The pH-sensitive system consisted of a monophosphorylated gemcitabine (p-GEM) and paclitaxel NP. As shown in the schematic of Fig. 3c, the study confirmed that the novel formulation (T-RKP) selectively disrupted α-SMA expression in the tumor core, destabilizing the internal core and eradicating tumor cells without affecting the outer stroma. Hence, this nanosystem enabled selective stromal targeting and improved drug efficiency and warrants further investigation to assess its therapeutic potential.

Apart from disrupting CAFs, another strategy consists in using cytotoxic drugs to directly target CAFs, which promote drug resistance in malignant cells by upregulating proteins like wnt16 and erecting physical barriers that hamper drug uptake and penetration [11]. For instance, Miao et al. [35] showed that Wnt16, upregulated in cisplatin-damaged CAFs, contributes to cisplatin resistance (Fig. 3d). Exposure to PEGylated cationic lipid corona (LCP) NPs carrying cisplatin stimulated Wnt16 secretion, leading to stroma reconstruction aggravated by drug resistance (Fig. 3d(i)). To downregulate and inhibit Wnt16, liposome-protamine-hyaluronic acid NPs carrying anti-Wnt16 siRNAs were tailored to enhance efficiency of cisplatin-containing LCP NPs within the stroma-rich environment of a bladder cancer model and foster cisplatin responses (Fig. 3d(ii)). The LCP NPs substantially enhanced antitumor responses, even in advanced stages of stroma-enriched bladder cancer. Moreover, mechanistic investigation revealed that Wnt16 downregulation elicited multiple beneficial effects by modulating paracrine interactions with neighboring cellular populations, resensitizing neoplastic cells to cisplatin, remodeling the TME and fibroblast populations, and inhibiting angiogenesis [35]. These results indicate that nano-based combination approaches can alleviate cisplatin resistance and resensitize the TME to cisplatin by inhibiting Wnt16 in damaged CAFs. Another study by the same group investigated the therapeutic effects of LCP NPs loaded with quercetin, a dietary flavonoid shown to inhibit Wnt16 in CAFs [37]. The NP formulation significantly downregulated Wnt16 and yielded synergistic anticancer action with cisplatin NPs in stroma-enriched bladder cancer. Quercetin NPs successfully remodeled the environment and enhanced the uptake of nano-based chemotherapeutics into tumor niches. These two innovative studies delineate novel mechanisms of CAF targeting and tumor drug resensitization in solid tumors that could successfully remodel the TME and improve anti-tumor effects.

To formulate novel nano-based approaches that overcome CAF-induced drug resistance, the Huang team devised an ingenious and innovative strategy aimed at turning the ‘foe’ into a ‘friend’ [36]. For this purpose, they tailored plasmids encoding TNF-related sTRAIL loaded into lipid-coated protamine DNA NPs and administered to xenograft models of desmoplastic bladder cancer (Fig. 3e(i and ii)). sTRAIL induced apoptotic death in malignant cell nests neighboring CAFs. It also reprogrammed CAFs into a quiescent state, thus ablating the tumor and reshaping the TME to enhance second wave nanotherapeutic treatment (Fig. 3e(iii)). The tested approach proved effective in a xenograft PC model, where dense stroma blocks the delivery of NPs [36]. These results demonstrate that NPs can successfully target and modify CAFs, thus offering a potentially effective approach for treating desmoplastic stroma-dense solid tumors. Other NP formulations designed to target CAFs have also been developed. For example, Ji et al. [38] designed an amphiphilic cleavable peptide that responds to fibroblast activation protein alpha (FAP-α), a protease on CAF surfaces. Cleavable amphiphilic peptide (CAP) monomers self-arranged in aqueous solutions due to their amphiphilic nature, while hydrophobic chemotherapeutic agents transformed these assemblies into spherical drug-loaded NPs. CAP-NP disassembly upon FAP-α cleavage efficiently released the encapsulated drug at neoplastic niches. This innovative Transformers-like strategy could effectively disturb the dense stromal barrier and improve drug accumulation, thus providing versatile solutions for antitumor therapy [38]. Another study reported a CAF-targeting nanoliposomes encapsulating navitoclax, which can specifically bind to the CAF protein tenascin C [39]. The navitoclax-loaded nanoliposomes showed improved cellular uptake and exerted higher cytotoxic effects. The nanoliposome formulation delivered navitoclax to CAFs both in vitro and in animal models, eradicated CAFs and suppressed tumor growth, indicating that this modality could serve as a potential tumor killing approach. Zen et al. [40] developed another innovative approach to improve CAF targeting and avoid toxic side effects. They engineered a ferritin NP altered with a specific single chain variable fragment and PS, which enable specific homing to CAFs and their eradication by photo-irradiation without damaging healthy tissue. Mechanism of action investigations revealed that the nano-photoimmunotherapy system suppressed C-X-C motif chemokine ligand 12 (CXCL12) and inhibited ECM deposition. Those cascades are modulated by CAFs in tumors, promote the exclusion of T cells, and prevent contact between tumor and T cells and efficient immune targeting of neoplastic cells [40]. By selectively targeting CAFs, the nano-photoimmunotherapy system reversed those effects and improved T cell infiltration, thus enhancing tumor suppression. This elegant study provides a proof-of-concept model of how selective nanotherapeutic solutions can reshape the TME to reactivate anti-tumor immunity. As these examples show, advances in nanotechnology can facilitate the development of novel modalities capable of reprogramming the TME to reactivate anti-tumor pathways, improve drug delivery and circumvent drug resistance. These novel strategies could pave the way for personalized treatment strategies and can be used in combination with current therapeutic regimens to enhance outcomes.

## Harnessing nanoparticles for targeting tumor physiology

### Nanoparticle strategies for modulating the tumor extracellular matrix

Metastasis involves the spread and development of a novel nidus at a distal niche distinct from the original tumor and is responsible for 90% of cancer deaths. The ECM, which contributes to tumor progression, can be exploited to prevent metastasis. Several studies have focused on developing ECM mimics using artificial molecules. Laminin is one of the key components of the neoplastic ECM. Hu et al. [41] developed a laminin-mimicking peptide for the construction of an artificial ECM aimed at preventing tumor invasion. The laminin-mimic was able to self-assemble and form NPs through hydrophobic interactions. The resulting NPs were converted into nanofibers upon binding with laminin receptors and integrins on tumor cells. The artificial ECM laminin NP mimics had a high retention time and accumulated at neoplastic sites for 3 days, successfully inhibiting metastasis in various solid tumor models. Another biomimetic strategy consisted in developing cell-adhesive patterns that simulate tumorous ECM with magnetic field-elicited assembly of magnetic nanocarriers on agarose hydrogel [42]. Driven by magnetostatic fields, the magnetic nanomaterials successfully assembled into both fibrous and mesh-like patterns, guided cell adhesion and migration and promoted the aggregation of osteoclasts [42]. As these studies demonstrate, biomimetic nanomaterials offer innovative solutions that can strengthen the ECM by modulating adhesion and metastasis.

Recent insights on the mechanics of native tumor ECM assembly have inspired novel approaches aimed at targeting various stages of ECM deposition to correct abnormal architectures. Grossman et al. [43] used advanced microscopy to monitor collagen assembly in a 3D matrix. They identified LOXL2 antibodies capable of modifying the width and natural alignment of endogenic collagen without interfering with ECM composition. Altered collagen morphologies disrupted adhesion and invasion in human bladder cancer (BC) cells. Furthermore, the inhibitory antibodies interfered with neoplastic collagen superstructure and reduced tumor growth in mice models [43]. These studies illustrate how nano-based mimetic materials and antibodies can be used to modify ECM architecture and shift the TME towards anti-metastatic and tumor suppressive phenotypes that support tumor eradication. As such, these modalities open new horizons of therapeutic and clinical intervention and usher in promising possibilities of personalized therapies.

### Nanoparticle strategies for targeting the tumor vasculature

**Fig. 4:**
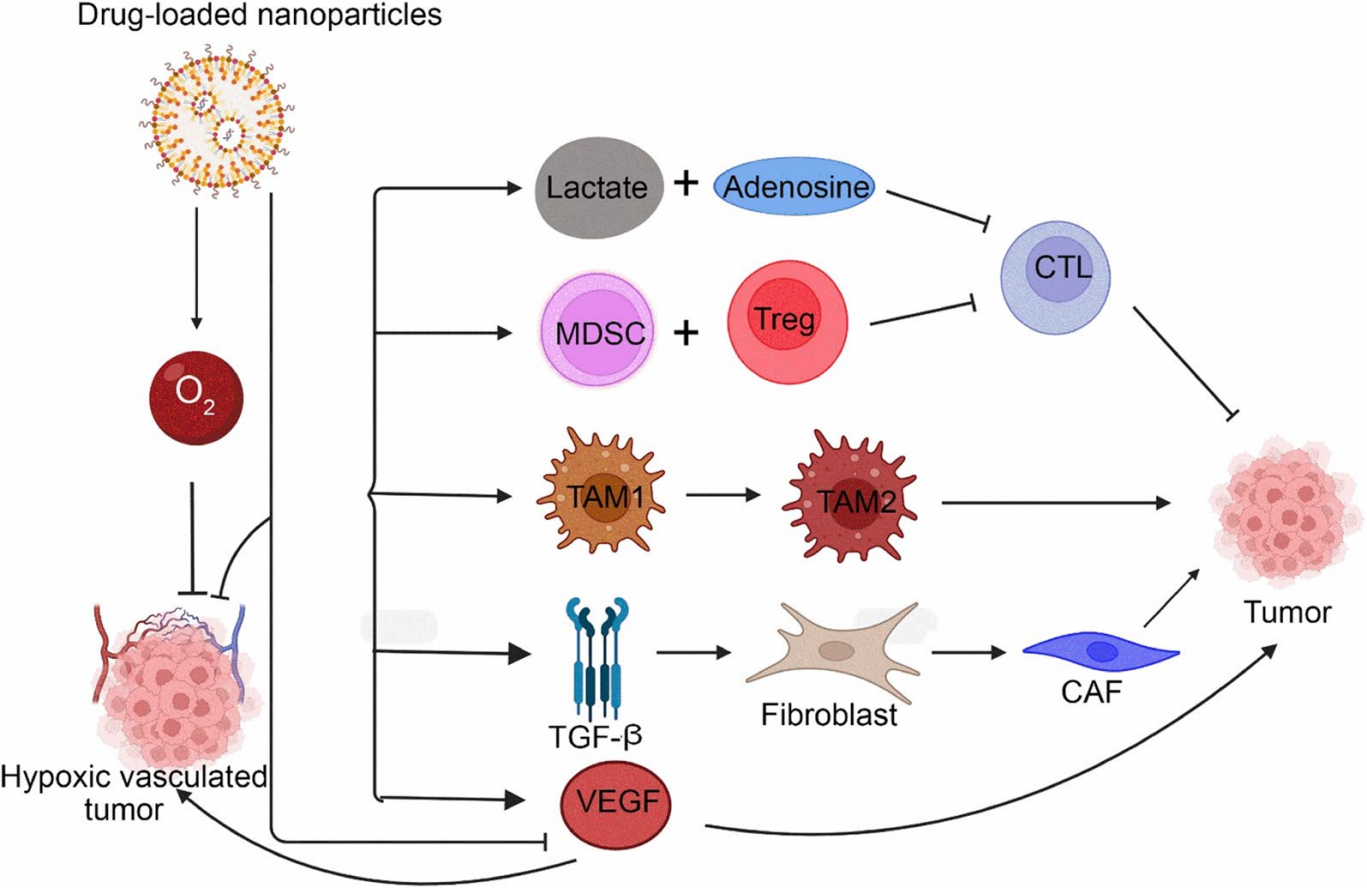
Drug loaded NPs can target the hypoxic tumor vasculature, VEGF and immune cell populations**:** Lactate and adenosine as well as MDSCs and Tregs inhibit cytotoxic T lymphocytes and drive tumor growth. The conversion of M_1_ macrophages to M_2_ macrophages also contributes to tumor growth. Additionally, TGFβ-induced fibroblasts activate CAFs, which promote tumor progression. VEGF stimulates abnormal tumor vasculature and cancer development. Drug loaded NPs can target all of these pathways to suppress tumor growth. Furthermore, NPs loaded with oxygen can inhibit the hypoxic tumor vasculature by inducing oxygen, thus reversing hypoxia and limiting tumor growth

Cancer vasculature is characterized by abnormal appearance and functions. Its impaired activity is aggravated by the hypoxic TME, which stimulates the production of angiogenic molecules such as TGF-β and VEGF. The mechanism is schematized in Fig. 4 [2]. Briefly, the exaggerated imbalance between anti-angiogenic and pro-angiogenic factors elicits rapid and anomalous vessel formation in tumor niches. These tortuous, unevenly distributed vessels exhibit increased permeability, leading to high protein leakiness in vessels and elevated interstitial fluid pressure in the TME. High interstitial fluid pressure further compresses blood vessels and exacerbates tumor hypoxia [2]. Dysfunctional vessels restrict T cell infiltration and hamper drug delivery, thus creating a protective barrier that shields the tumor and obstructs therapeutic responses [2]. Targeting the tumor vasculature, which meets the high nutrition demands required for uncontrolled tumor cell growth, is a key strategy to remodel and reshape tumor responses. Disrupting the aberrant tumor vasculature can result in insufficient blood and nutrition supply to cancer sites, provoking necrosis and subsequent apoptosis of malignant cells [10]. Although vasculature normalization is a theoretically sound approach, its actual effects have been thwarted by acquired endothelial resistance [2]. In fact, anti-angiogenic therapy reverses aberrant vasculature only transiently but does not alter it. This “transient” vessels normalization has been termed “normalization window” and is critical for anti-tumor therapy [2]. NPs have recently emerged as a potential strategy for delivering anti-angiogenic that target the vasculature while obviating the need to determine for the “normalization window”.

**Fig. 5:**
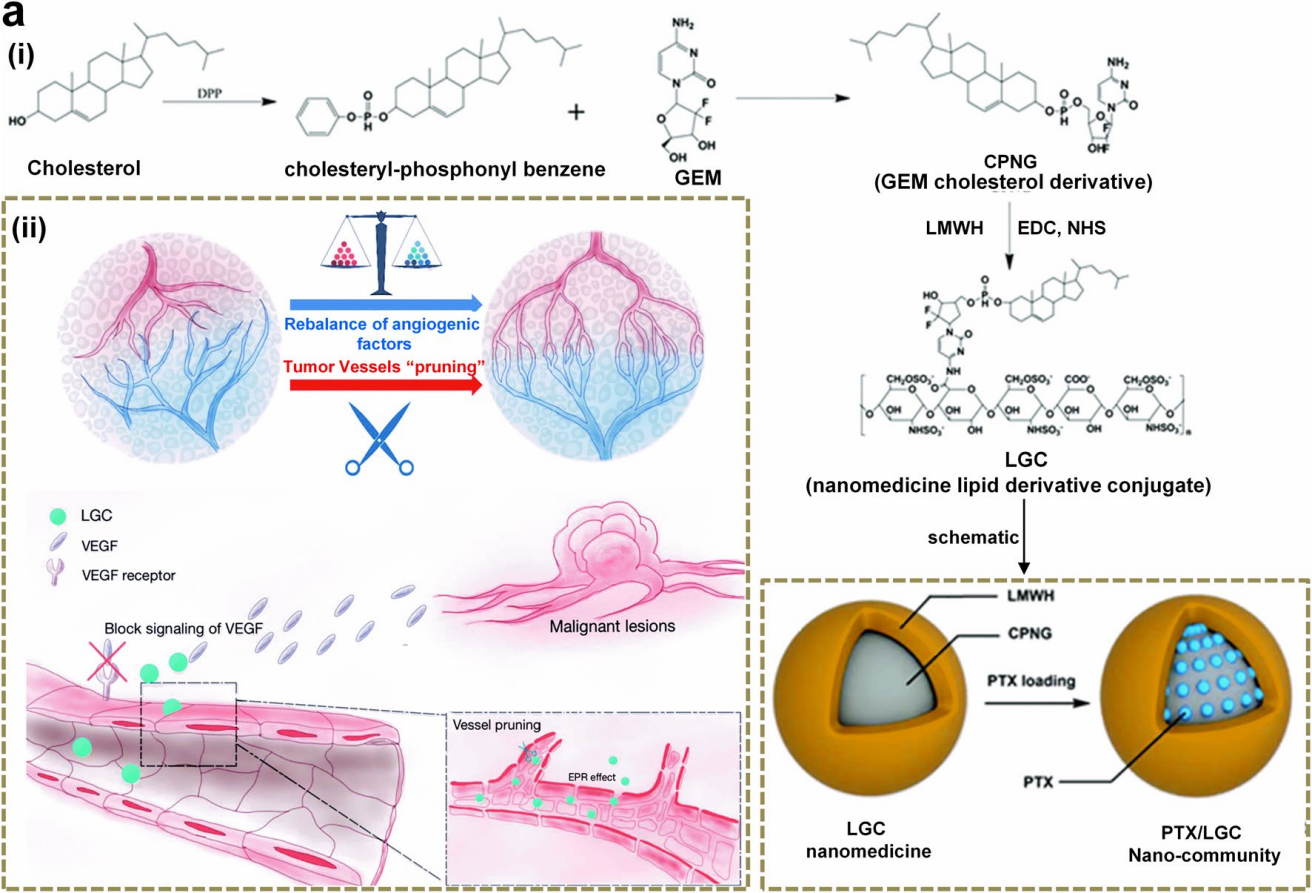
Vasculature normalization using nanomedicine: (i) synthetic reaction mechanism for the development of PTX/LGC nano-community and related schematic illustration, and (ii) tumor vasculature normalization mechanism by PTX/LGC nano-community via two different approaches (a) pruning and (b) blocking of VEGF pathways. Copyright 2019 by Du [45]

Building on this concept, Zhou et al. [44] devised a polymeric NP for sequential controlled delivery of DOX and the EGFR inhibitor erlotinib Ei to enhance therapeutic responses in TNBC. This novel NP can sequentially release Ei then DOX to sensitize tumor cells to DNA damage by DOX. The system achieved elevated tumor accumulation and improved therapeutic responses, indicating that this sequential model might represent an elegant method for targeting neoplastic cells. Another group, Du et al. [45], devised a tumor vasculature normalizing approach using nano-lipid derivative conjugates as shown in Fig. 5a, which consists of anti-angiogenic molecules coupled with GEM plus low molecular weight heparin (LMWH) (Fig. 5a(i)). This nanostrategy exploits anti-VEGF approaches and metronomic chemotherapy to enhance tumor vasculature normalizing effects (Fig. 5a(ii)). The researchers generated a “nano-community” though loading cytotoxic agents including paclitaxel into lipid derivative conjugates to repair tumor vasculature and deliver the drug load (Fig. 5a(i)), thus improving therapeutic outcomes while obviating the necessity of identifying the exact normalization window [45]. This study demonstrates that nanotherapy can be combined with tumor vasculature normalization approaches to improve the delivery of chemotherapeutic agents into solid tumors [45]. Hence, nano-based solutions can address therapeutic challenges in novel ways, enabling effective reprogramming of the TME. Accordingly, novel findings suggest that simultaneous normalization and targeting of the tumor vasculature and ECM, as opposed to anti-angiogenesis alone, can refine the efficacy of nanotherapeutics [46]. Li et al. [47] devised a novel platform for facilitating tumor vasculature normalization. They used gold NPs as a carrier for delivering recombinant endostatin, which inhibits angiogenesis and lymphatic metastasis by reducing VEGF levels and gene expression. The gold NPs improved accumulation of Recombinant human endostatin in tumor sites, promoted transient vessel normalization in xenografts, decreased hypoxia and leaking and improved therapeutic responses. Ultimately, refining the size of vasculature-targeting NPs will greatly improve drug delivery in solid tumors.

**Fig. 6:**
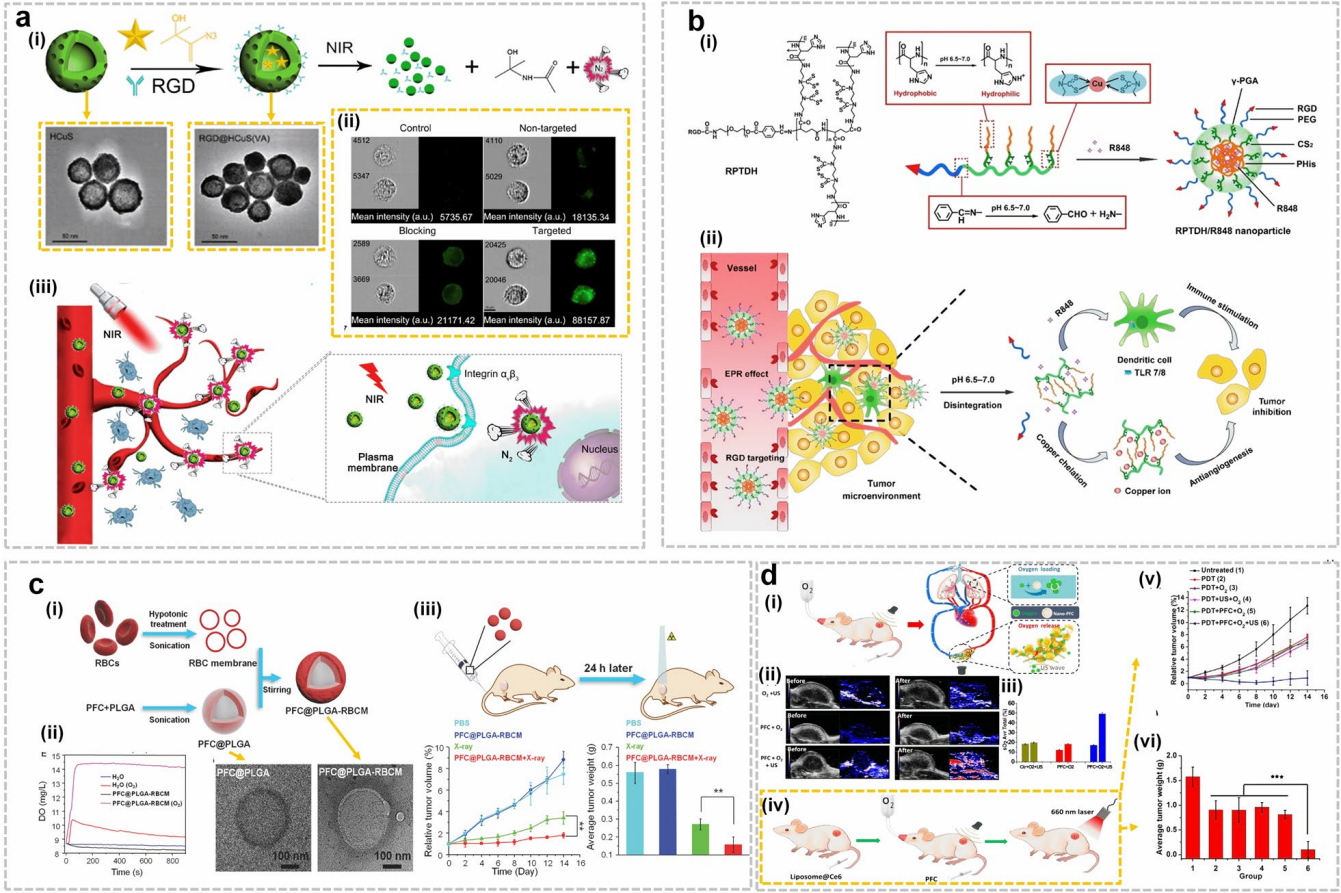
Disrupting the tumor vasculature using VDAs: **a** (i) Schematic illustrates the synthesis of RGD@HCuS with N_2_ bubble generation and corresponding TEM images of HCuS nanoparticles and RGD@HCuS nanomedicine.(ii) intra cellular localization of RGD@HCuS nanomedicine and (iii) targeted mechanism for the tumar vasculature destruction using NIR laser activated bomb-like effect. Copyright 2019 by Gao [48]. **b** (i) Synthesis approach of RPTDH/R848 nanoparticles and (ii) schematic establishment of the mechanism involved in suppression of growth and metastasis in breast cancer cells. Copyright 2019 by Zhou [51]. **c** (i) Schematic representation of the development of artificial RBCM coated PFC@PLGA nanoparticles and corresponding TEM images of PFC@PLGA and PFC@PLGA@RBCM nanoparticles, (ii) quantification of oxygen from the PFC@PLGA@RBCM nanoparticles added water with and without applying the pressure and (iii) in vivo radiotherapy and corresponding effect on the tumor growth. Copyright 2017 by Gao [52]. **d** (i) Schematic shows the ultrasound induced local oxygenation with nano-PFC, (ii) photoacoustic imaging established oxygenated and deoxygenated hemoglobin by irradiating with 850 and 750 nm, respectively, (iii)oxyhemoglobin quantification in the tumor, (iv) schematic representation of the ultrasound activated nano-PFCs generated tumor oxygenation enhanced PDT, (v) tumor growth curves and (vi) corresponding tumor weights after different PDT treatments. Copyright 2016 by Song [53]

Disrupting the tumor vasculature using vasculature disrupting agents (VDAs), which interfere with cancer blood vessels and reduce blood supply to the tumor center, is also a viable strategy for attacking tumor growth [2]. To test this strategy, a near infrared (NIR)-laser-induced ‘nanobomb’ (Fig. 6a) was designed as a non-invasive targeted approach for disrupting neoplastic vasculature in an expeditious and accurate fashion [48]. The nanobomb was fabricated by encapsulating vinyl azide into functionalized c(RGDfE) hollow CuS (HCuS) NPs (Fig. 6a(i)). Figure 6a(ii) confirmed that the obtained RGD@HCuS was selectively and efficiently internalized into α_v_β_3_-positive endothelial cells within the tumor vasculature. Following NIR radiation, N_2_ bubbles were released rapidly due to increased local temperatures. Released N_2_ bubbles instantaneously exploded in the TME (Fig. 6a(iii)), thus destroying the neovasculature and inducing the necrosis of surround cancer cells [48]. Hence, this nanobomb might be a candidate for clinical translation for GI cancers. Additionally, nano-based vessel disrupting solutions might prove efficient in the context of combination therapies, in synergy with radio or immune therapy. For instance, Satterlee et al. [49] devised a combination strategy aimed at slowing growth stroma-enriched tumors. The approach aimed at eliciting apoptosis in the center while increasing NP uptake using CA4P (VDA) to improve accumulation of radiation-carrying NPs for RT. The resulting platform prolonged the apoptosis of tumor cells, decreased fibroblast density and improved treatment with another NP solution containing cisplatin [49]. Additionally, VDAs have been tested in combination with immunotherapeutic agents. For instance, Set et al. [50] used guardiquimod (TLR7/8 agonist) encapsulated PLGA NPs (Gardi-PLGA) as a delivery carrier for gardiquimod in combination with the VDA DMXAA to analyze BMDC activation. Interestingly, both DMXAA and Gardi-PLGA failed to elicit cancer killing in melanoma cells. However, the combination regimen significantly reduced tumor volume. This effect could be attributed to the immune-stimulating effects of Gardi-PLGA coupled with the vasculature-disrupting activity of DMXAA, which synergistically target DCs and the tumor [50]. Hence, the combination of TLR agonists and VDAs might be an effective strategy for treating solid tumors in humans. Other approaches combining anti-angiogenic agents and immunotherapy include a study by Zhou et al. [51] who used antiangiogenic copper.

chelating polymers to fabricate NPs loaded with the TLR7/8 agonist resiquimod (R848). The corresponding synthesis approach is presented in Fig. 6b(i). The combination therapy dramatically suppressed growth and metastasis in breast cancer cells through R848-elicited immune activation and copper-deficiency induced anti-angiogenesis (mechanism schematized in Fig. 6b(ii) [51]). Another anti-angiogenic strategy developed by Sun et al. [54] consisted of co-delivering Cu(I) chelators coupled with chemotherapeutics that can target newborn vessels. The synthesized micelle was co-loaded with DOX plus Probe X, which can switch from OFF to ON to indicate Cu^+^ capture through NIR and photoacoustic signals. Co-delivery of antiangiogenic and chemotherapeutic reagents successfully suppressed the tumor with low toxicity, thus providing insights on the design and development of sophisticated theranostic platforms that can overcome the limitations of conventional therapy. In summary, strategies combining vasculature-targeting agents and immunotherapy warrant further development and investigation. Future studies will establish the feasibility and efficiency of these approaches in human patients.

## Chemical modulation of the TME using nanoparticles

### Nanoparticle strategies for targeting tumor hypoxia

Targeting the hypoxic TME remains one of the main frontiers in cancer treatment. During tumor development, rapid replication and growth of cancer cells and aberrant blood vasculature results in a reduced insufficient oxygen supply [2]. Poor TME oxygenation results in hypoxia and a cascade of events promoting tumor development, angiogenesis and subsequent metastasis. Hypoxia causes TME immunosuppression by upregulating CCL22-28, activating Tregs and MDSCS and polarizing macrophages towards M_2_ phenotypes [55–58]. Hypoxia also increases immunosuppressive signals such as TGF-β and VEGF and levels of PDL-1 expression on tumor and immune cells [2]. These phenomena contribute to drug resistance, particularly to radiotherapy which requires oxygen molecules for tumor cell eradication. Due to the wide-ranging tumorigenic effects of hypoxia, it has become the focus of various cancer-eradicating therapeutic strategies. Many nano-based solutions have been developed to target hypoxia, including (1) oxygen delivery to hypoxic tumors, (2) oxygen generation in hypoxic tumors and (3) directly targeting hypoxia [11]. The following sections will explore some of the therapeutic and technical advances in this field.

#### Oxygen delivery to hypoxic tissue

Direct oxygen delivery to hypoxic tumor environments represents a promising strategy to replenish oxygen in the TME. As such, it necessitates the selection of adequate oxygen carriers, such as perfluorocarbon (PFC), utilized as a reservoir for transporting oxygen to hypoxic milieus. PFC-based nanotherapeutics have been tested as therapeutic strategies for oxygen replenishment in cancer. Gao et al. [52] designed an artificial nanosized red blood cell (RBC) platform (Fig. 6c) by encapsulating the blood substituent PFC within PLGA to construct PFC@PLGA NPs coated with RBC membrane (RBCM), which is presented in Fig. 6c(i). The resulting PFC@PLGA-RBCM NPs with PFC cores displayed efficient oxygen loading capacity and prolonged blood circulation time (Fig. 6c(ii)) because of RBCM coating. The in vivo studies (Fig. 6c(iii)) confirmed that the NPs successfully delivered O_2_ to tumors following intravenous injection, relieved hypoxia and enhanced the efficacy of radiation therapy. This unique nano-based RBC mimic, which efficiently delivers oxygen to tumors and supports RT treatment, warrants further investigation to establish its clinical efficacy. Song et al. [53] further fine-tuned this concept by developing a platform that modulates the hypoxic TME using nano-PFC oxygen shuttles to power ultrasound-based oxygen delivery to tumors as shown in Fig. 6d(i). They injected PFC nanodroplets stabilized by albumin into cancer-bearing mice subjected to hypoxic breathing. Figure 6d(ii and iii) established that, PFC nanodroplets can rapidly release O_2_ into tumor tissue and recirculate into the lung for additional reoxygenation under ultrasound stimulation. Repeated cycles of ultrasound stimulation of PFC nanodroplets could thus dramatically improve tumor reoxygenation and boost therapeutic outcomes for both radiotherapy (RT) and photodynamic therapy (PDT), as shown in Fig. 6d(iv–vi) [53]. This simple yet efficacious strategy might thus overcome hypoxia-related drug resistance in cancer therapy. A similar strategy consisted in loading Bi_2_Se_3_ NPs with PFC as oxygen carriers and using near-infrared light to induce burst oxygen release from NPs, thus overcoming hypoxia-related RT-resistance [59]. The same group developed another nanoplatform aimed at resensitizing tumor cells to RT through improving oxygenation. They constructed PEG-stabilized PFC nanoscale droplets decorated using TaOx NPs as RT sensitizers [60]. The TaOX@PFC-PEG NPs function on two fronts: (1) they absorb X-Ray through TaOx and concentrate this radiation energy in cancerous cells; (2) PFC functions as an O_2_ reservoir that can release oxygen gradually to enhance tumor oxygenation. This elegant nanoplatform improved RT treatment in mouse models and achieved efficient and desirable therapeutic outcomes with reduced toxicity [60]. In more recent work, Liu et al. [61] developed a novel on-demand O_2_ releasing NP triggered by NIR light to achieve synergistic photodynamic/photothermal therapy. This smart nanoplatform displayed both deep intratumor penetration and high tumor accumulation rates. It also achieved continuous, on-demand complete oxygen release to relieve hypoxia during phototherapy and allowed imaging-guided dual-model cancer therapy [61]. This work could thus serve as a proof-of-concept model for designing nano-solutions with prolonged oxygen release that enable synergistic phototherapy in hypoxic tumors.

#### Generating oxygen in hypoxic tumors

**Fig. 7:**
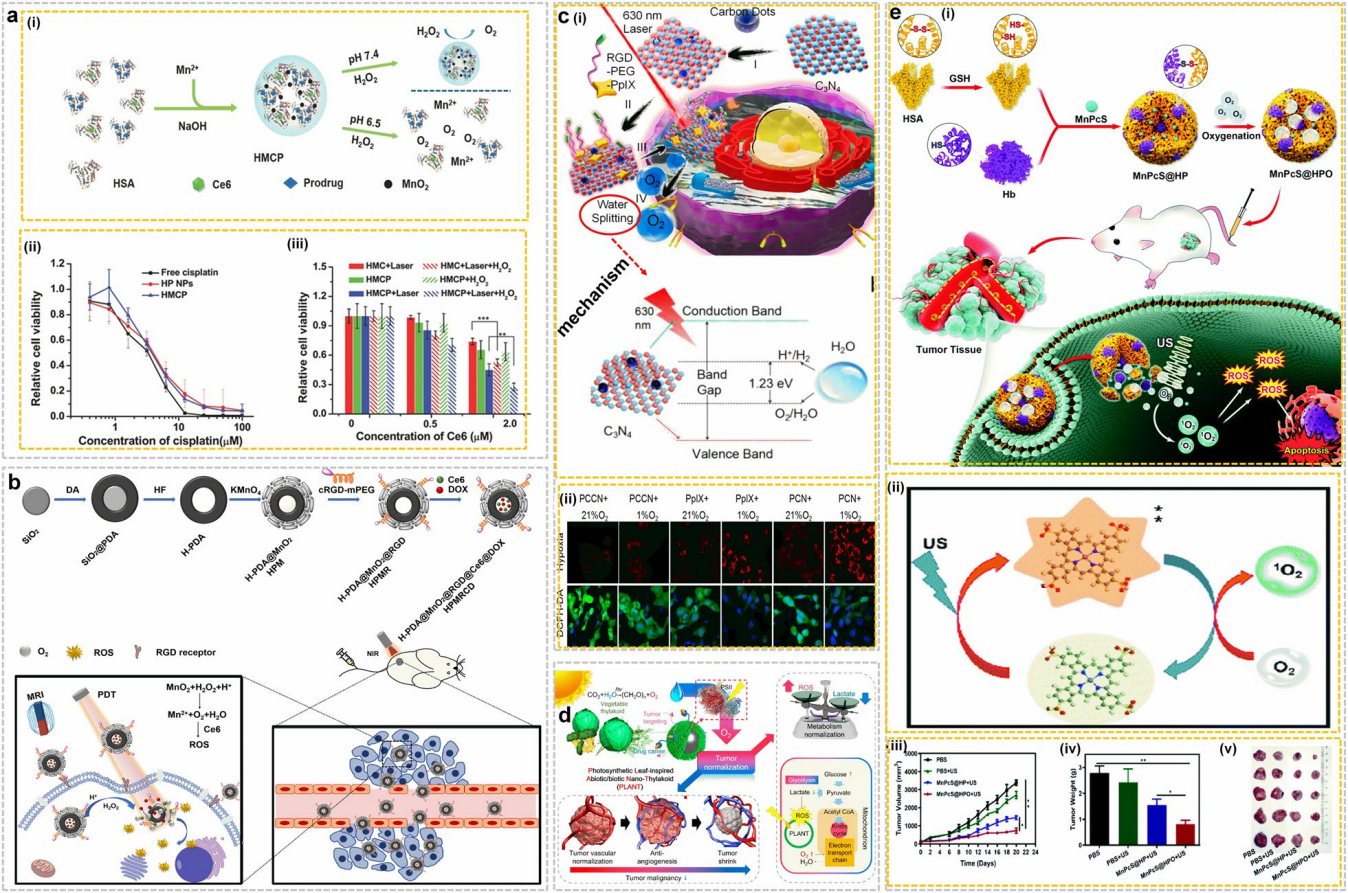
Reprogramming the TMEs by alleviating hypoxia: **a** (i) Schematic represents the development of albumin-decorated MnO_2_ nanoparticles with Ce6 and Pt conjugation (HMCP) and simultaneous production of oxygen generation H_2_O_2_ solution, (ii) cell viability after treating with HMCP nanoparticles, and (iii) cell viability after PDT (irradiation with 660 nm) with and without addition of H_2_O_2_. Copyright 2016 by Chen [62]. **b** Schematic represents the development of HPMRCD and its potential application mechanism in MRI imaging, chemophotodynamic therapy. Copyright 2021 by Wang [63]. **c** (i) Schematic representation of the synthesis of carbon dot doped C_3_N_4_ and PCCN and corresponding PDT enhanced by 630 nm light driven water splitting and corresponding water splitting mechanism, (ii)quantification of PCCN induced oxygen production, hypoxia reversion and ROS generation. Copyright 2016 by Zheng [65]. **d** Schematic shows the PLANT system induced tumor normalization. Copyright 2018 by Zheng [66]. **e** (i) Illustration of the development of MnPcS@HPO NPs and corresponding SDT antitumor activity, (ii) mechanism involved in the conversion of O_2_ to ^1^O_2,_ and (iii-v) tumor volume, weight and corresponding photograph images after SDT. Copyright 2022 by Yin [68]

While direct oxygen delivery to tumors represents a theoretically easy strategy to relieve hypoxia, nanosystems for efficient oxygen loading remain limited and could provoke nanocarrier-related toxicity. An alternative approach is to specifically deliver oxygen-producing agents, such as metformin, MnO_2_ or catalase, that induce chemical reactions leading to oxygenation in tumors [11]. Among these various nanocarriers, MnO_2_ and catalase-based nanosolutions represent common delivery platforms for oxygen generation through H_2_O_2_ catalysis at tumor sites [11]. Based on this concept, Chen et al. [62] developed an innovative pH/H_2_O_2_ dually responsive and smart nano-delivery platform fabricated using albumin-decorated MnO_2_ (Fig. 7a(i)) and capable of reprogramming the TME by alleviating hypoxia. This dual system synergistically increased the efficacy of both chemotherapy and PDT by elevating oxygen levels at hypoxic tumor sites (Fig. 7a(ii and iii)), thus significantly inhibiting tumor growth in mouse models. Recent work by Wang et al. [63] exploited the potential of MnO_2_ based NPs to boost chemo-photodynamic therapy. The group manufactured a theranostic nanoplatform using hollow polydopamine (HPDA) nanostructures decorated with MnO_2_ for drug delivery (HPMRCD), as shown in Fig. 7b. Upon reaching tumor sites through active targeting using RGD, the MnO_2_ shell and PDA of HPMRCD can decompose and release DOX and Ce6 in the TME (Fig. 7b). MnO_2_ can interact with H_2_O_2_ to generate O_2_, thus boosting the efficiency of PDT [63]. Hence, this all-in-one platform might represent a promising approach for chemo-photodynamic therapy in hypoxic tumors. Recent work by Li et al. [64] also utilized MnO_2_ NPs to devise a multifunctional platform to enhance photothermal therapy (PTT)/PDT treatment. This platform consisted of g-C_3_N_4_ loaded with MnO_2_ and CuS NPs. The resulting F127@CNS-CuS/MnO_2_ successfully overcame the limitations of single mode tumor therapy and potentiated PDT/PTT treatment. The MnO_2_ particles generated oxygen to attenuate hypoxia in the TME and reacted with overexpressed glutathione in cancer cells, further enhancing therapeutic effects [64]. This multifunctional nanoplatform could be employed as a tool to enhance synergistic PDT and PTT treatment in hypoxic tumors, thus representing a potentially promising companion solution.

Other innovative approaches include oxygenation strategies based on light-mediated water splitting. For instance, Zheng et al. [65] developed a carbon-dot-doped C_3_N_4_ nanocomposite (PCCN) to enable light-driven H_2_O splitting (Fig. 7c(i)). PCCN increased oxygen levels and ROS generation in hypoxic environments following light irradiation, as quantified in Fig. 7c(ii). Additionally, PCCN reversed hypoxia-induced PDT resistance and reduced tumor growth, which is shown in Fig. 7c(ii) [65]. These results indicate that strategies based on water splitting might improve intratumoral O_2_ levels and resensitize hypoxic tumors to PDT. Other innovative technical approaches include biomimetic nanosystems. For example, Zheng et al. [66] drew inspiration from plant biomimetics to develop a novel oxygen-generating nano-solution, as shown in Fig. 7d. They designed a leaf-inspired biotic/abiotic nano-thylakoid photosynthetic (PLANT) platform fusing thylakoid membranes with NPs for O_2_ generation. This PLANT platform successfully increased oxygen generation and suppressed anaerobic respiration in tumor spheroid models. PLANT normalized the metabolic network and adjusted the aberrant structure and activity of tumor vessels and enhanced the efficiency of phototherapy and anti-angiogenesis approaches in tumor models [66]. Hence, this plant-inspired approach could represent an effective and easy strategy for normalizing oxygen levels in hypoxic tumors. Another recent study by Tao et al. [67] proposes a multifunctional nanoplatform (hollow black gold-O_2_-C labeled as HABT-C) displaying multienzyme mimetic properties for sonodynamic therapy (SDT) to relieve tumor hypoxia and overcome tumor immunosuppression. HABT-C can initiate a cascade reaction that generates oxygen to alleviate hypoxia in tumor sites and provides sufficient oxygen to amplify ROS production leading to apoptosis. Additionally, TiO_2_ comprises electron-enriched sites that reduce the adsorption energies of surface O_2_ and H_2_O and facilitate ROS generation and oxidative damage in cancerous cells. In mouse models, HABT-C@HA showed satisfactory anti-tumor activity [67]. The multifunctional system induced immune responses following UV irradiation and improved the infiltration of immune cells into tumor niches. Hence, this study illustrates how novel nano-based approaches can be exploited to develop platforms capable of reversing hypoxia through direct physical oxygen generation in hypoxic niches while reprogramming the TME and enhancing immune responses. New work by Yin et al. [68] also reported a novel oxygen-producing nanoplatform aimed at improving SDT (Fig. 7e). The group developed an oxygen-enhanced nanosensitizer platform (MnPcS@HPO) fabricated using human serum albumin (HSA) and hemoglobin (Hb) by disulfide reconfiguration (Fig. 7e(i)). MnPcS were then encapsulated to generate self-supplementing O_2_ NPs capable of potentiating SDT (Fig. 7e(ii)). Harnessing the O_2_-carrying abilities of Hb coupled to HSA’s tumor-targeting properties, the MnPcS@HPO NPs successfully targeted cancerous niches, relieved hypoxia and improved SDT anticancer activity (Fig. 7e(iii-v)) [68]. Ding et al. [69] also developed a nano-based solution aimed at alleviating hypoxia and synergizing chemo-photodynamic therapy. The group designed an RBC biomimetic theranostic NP SPN-Hb@RBCM characterized by improved photostability, PSs accumulation, and O_2_ self-supply capacity to increase PDT efficacy following NIR laser irradiation. The conjugated Hb served as an oxygen transporter to reverse tumor hypoxia and resensitized the cells to PDT treatment [69]. This study offers valuable insights into biomimetic and theranostic nanoplatforms capable of enhancing oxygenation and synergistic PDT/CDT therapy against hypoxic cancers. Other recent hypoxia-relieving nanosystems include a metformin-based nano-reactor [70], catalase nanocrystals carrying methylene blue to serve as an oxygen supplied and guided platform for PDT therapy [71], and CeO_2_ quantum dots anchored to MnO_2_ nanoflowers [72]. As these various studies show, advances in nanotechnology can be harnessed to develop practical solutions that address key challenges posed by the tumor microenvironment, such as tumor hypoxia. Innovative nanoscale platforms can deliver oxygen directly to hypoxic niches or elicit oxygen self-generation in anaerobic neoplastic sites, thus physically reshaping neoplastic environments and reactivating anti-tumor phenotypes. Future work will determine if these theranostic solutions can be successfully translated from pre-clinical models to the clinical setting to serve as companion therapeutic tools in combination with existing treatment modalities such chemotherapy, radiation therapy or photodynamic regimens.

### GSH, ROS, and ATP-responsive nanoparticle approaches

Elevated ROS levels represent a hallmark of neoplastic cells and tissue. Although aberrant redox status promotes cancer development, it can be harnessed to potentiate treatment. ROS accumulation in neoplastic milieus results in oxidative stress, leading to increased levels of ROS scavengers such as glutathione (GSH) [73]. Elevated ROS and GSH provide avenues for designing novel nanotheranostic strategies aimed at treating cancerous lesions by leveraging abnormalities characteristic of tumor growth and biology using specifically engineered tools. The rationale behind redox-based nanotherapeutic approaches is the increased sensitivity of cancerous cells to altered ROS levels, which can be exploited to improve pre-existing therapeutic approaches such as chemotherapy or immunotherapy [73]. For instance, redox-sensitive nanosolutions take advantage of the increased GSH levels in tumor cells to refine therapeutic intervention strategies. Redox moieties, namely disulfide bonds, endow NPs with improved drug release capacity and cytotoxicity [73]. This property has enabled the development of redox sensitive polymeric micelles or NPs that trigger drug release at elevated GSH levels and enhance the efficacy of chemotherapeutic agents such as docetaxel [74] or paclitaxel [75] in vitro and in mouse models.

To engineer a new spatiotemporal and chemotherapeutic nano-based application, Iyer et al. [76] developed GSH-responsive, polyurethane NPs (GPUs) comprising GSH cleavable DS bonds that respond to high GSH levels in lung tumor cells. Cisplatin loaded GPUs (CGPUs) successfully localized to lung tumor sites and exhibited dose-dependent cisplatin release, leading to the inhibition of tumor growth. Hence, these GSH-responsive and biodegradable NPs could be utilized for on-demand chemodrug release to improve chemotherapeutic outcomes. Liu et al. [77] formulated a GSH-sensitive prodrug NP through complexation between a prodrug carrying a disulfide bond that contains camptothecin (G) and GalP5. The customized prodrug NPs were stable and efficiently triggered drug release in tumor milieus exhibiting elevated GSH concentrations. Studies also revealed that the engineered NPs selectively entered HepG2 cells overexpressing the asialoglycoprotein receptor due to active targeting action of galactose, which enhanced antitumor efficacy while reducing undesirable side effects. Another group designed a new pH/GSH responsive chitosan NP prepared through self-assembly and self-crosslinking strategy to improve PDT [78]. This carrier exhibits enhanced chemical stability as compared to classical photosensitizers and selective targeted release. The engineered SA-CS-NAC@IGC NPs enabled controlled release of NPs in the elevated GSH and low pH TMEs of tumor cells. Upon exposure to laser irradiation, the NPs produced high amounts of ROS, resulting in improved tumor inhibition. As these studies show, NPs can utilize and overcome the pathological biochemical properties of the cancerous TME, leading to improved selective drug targeting and therapeutic efficacy. Sun et al. [79] developed a symmetrical drug-dye conjugation (DDC) prodrug with a disulfide bond as a biochemical trigger. This versatile theranostic system has several useful applications. For instance, the NP can be selectively and actively targeted to tumorous niches using PEG-PLGA micelles. Elevated concentrations of GSH in the TME induce the cleavage of the disulfide bond, leading to DDC decomposition, wherein both dye and drug are simultaneously released in one-to-one controlled mode. After disintegration, the turned-on probe can emit NIR fluorescence, which allows accurate non-invasive monitoring of drug distribution. Additionally, the dye, which serves as a photothermic sensitizer, can facilitate drug penetration into tumor sites, thus enhancing therapeutic effects. This “baby-sitting” approach offers new and valuable insights for developing versatile multimodal theranostic platforms that can aid in both monitoring and targeting drugs to cancer tissues and provides an alternative clinical strategy to hyperthermia.

Nano-based engineering strategies have also been used to develop ROS-responsive NPs aimed at targeting solid tumors and resensitizing cells to conventional treatment regimens. Zhang et al. developed Fenton reaction stimulating NPs containing ROS sensitive switches aimed at targeting metastatic TNBC through ROS amplification. The obtained P@P/H NPs elicited markedly high ROS levels and induced the expression of caspases 9 and 3 and cytochorome c and the inhibition of MMP-9 [80]. The subsequent cascade resulted in improved tumor inhibition rates, suggesting that this strategy could represent a useful solution for targeting resistant metastatic niches in breast cancer. Ma et al. [81] constructed a novel ROS- responsive UA-based dimer prodrug by joining two UA molecules through ROS cleavable linkage. This delivery system is characterized by its dimeric prodrug core that can selectively convert into drug molecules in the presence of ROS. This UA-NP was tested against GC tumors and shown to enhance anti-tumor effects in vitro and in animal models. ROS responsive NPs have also been used to potentiate anti-tumor responses to chemotherapy and PDT. For instance, a novel iRGD-BDOX@CPNS NP consisting of hydrophobic cores made of PVF chains that tightly enclose the BDOX prodrug resulted in efficient controlled drug release [82]. PFV can induce high levels of ROS through light triggering, leading to BDOX prodrug activation and drug release [82]. This elegant system can also enable drug delivery monitoring, thus providing valuable insights for tumor precision treatment and personalized medicine approaches. Banstola et al. [83] constructed an interesting novel nano-based ROS system. They synthesized a PDL-1 cancer cell and ROS sensitive dual targeted TMZ nanosystem designed to enhance therapeutic efficacy in hypoxic TME. Elevated ROS concentrations accelerated TMZ release from anti-PDL1-TKNPs. TMZ-mediated ROS hyperaccumulation provoked oxidative damage, resulting in mitochondria-elicited apoptosis. This innovative anti-PD-L1 TMZ-TK NPs amplified anti-tumor effects, including DNA damage and downregulation of angiogenic markers, thus offering a broad range of anti-tumor applications.

NP strategies that combine both ROS and GSH sensitivity have also been developed and tested in conjunction with pre-existing therapies. For instance, Zhu et al. [84] engineered GSH/ROS dual-sensitive supramolecular NPs (GOx@BNPs) that were activated by overexpressed ROS and GSH in the TME, thus accelerating NP dissociation, release capacity and glucose conversion into (.OH) through Fenton reactions. The anti-tumor effects of the dual system were mediated by high ROS, decreased ATP, MTP and apoptosis [84], suggesting that dual-sensitive supramolecular strategies offer a potential platform for synergistic interactions between starvation therapy, chemotherapy and chemodynamic therapy. Another dual ROS/GSH platform, PEG-PPS-GSNO@DOX NP, was developed to overcome multidrug resistance [85]. The resulting NPs exhibited high nitric oxide capacity facilitated by ROS-induced DOX release and GSH-mediated nitric oxide release. Nitric oxide reversed chemoresistance in vitro and increased DOX accumulation with minimal side effects in healthy cells, highlighting the promising potential of dual ROS/GSH NPs for cancer treatment.

In recent years, DNA nanotechnology has been harnessed to develop self-assembling NPs to enhance drug delivery and efficacy. DNA nanostructures can be engineered with customized shape, function and surface chemistries using various optimization strategies. For instance, Abnous et al. [86] developed a simple and elegant DNA NP consisting of two sequences: sequence 1 made of AS1411 aptamers and ATP; and sequence 2 containing antimiR-21 for co-delivery of antiapoptotic agents antimir-21 and KLA peptide. The NP was ATP-responsive and led to DNA nanostructure disassembly at high ATP concentrations and restoration of antimir-21 action, thus achieving enhanced inhibition of tumor growth in breast cancer cells. In another application, Liao et al. synthesized stimulus sensitive DNA microcapsules carrying different payloads and capable of dissociating through formation of aptamer and ligand complexes. The NPs were composed of ATP aptamer DNA coated microcapsules loaded with TMR-D, QDs or MP-11. In the presence of high ATP concentrations, the microcapsules dissociate by forming aptamer-ATP complexes, which leads to the controlled release of the drug loads. Although the obtained microcapsules exhibit selective delivery, their design process remains complicated and requires further refinement to improve its biochemical properties [87]. Cozzoli et al. [88] also reported the development of versatile and innovative DNA G-quadruplex micelle structures. The group conjugated lipid to DNA and assembled DNA headgroups into quadruplex structures, essential for micelle formation and stability. A hairpin DNA consisting of an ATP recognition site and a responsive domain acted as a complimentary sequence. When exposed to ATP, the aptamer attached to target molecules, causing rearrangement of the hairpin structure and subsequent exposure of the response domain, which can hybridize with G-4 micelles. This in turn caused micelle destabilization and cargo release. Hence, the structural rearrangements of the micelle can facilitate controlled drug release and enhanced therapeutic efficacy. As these various examples show, DNA-based NPs represent a budding field of research with promising applications for cancer treatment and personalized medicine. Further research is required to assess its efficacy and safety in human patients.

## Conclusion and future directions

The past decade of cancer research has unraveled the critical implication of the TME in cancer development and drug resistance, highlighting its importance as a key therapeutic target. Increased understanding of cancer genesis has revealed that tumors are not simply localized diseases but form a part of an entangled adaptive system marked by inflammation, metabolic and genetic disorders. Tumor eradication therefore requires successful reprogramming of the tumor microenvironment to resensitize neoplastic niches to existing or novel treatment modalities. Recent advances have allowed the design of nano-based therapeutic solutions tailored to target various aspects of the inflamed treatment-refractory TME, such as hypoxia, aberrant vascularization and CAFs. These nanotherapeutics modulate the TME through disruption and normalization. Despite those remarkable technological advances, however, most of the nanoplatforms discussed in this review are still in the initial design and optimization phases and have not yet been tested in human patients. One of the biggest challenges facing the field of TME-targeting nanotherapeutics is translation into the clinic, which will ultimately determine the validity and feasibility of the tested approaches. Improved knowledge of the molecular mechanisms driving TME dysregulation and their link to various stages of cancer progression will guide the design of more efficient nano-based solutions with improved targeting capacities.

## Data Availability

Not applicable.

## Abbreviations

α-SMA: α-Smooth muscle actin
APC: Antigen-presenting cell
BC: Bladder Cancer
BTK: Bruton Tyrosine Kinase
CAF: Cancer-associated-fibroblasts
CAP: Cleavable amphiphilic peptide
CAR-T: Chimeric antigen receptor T cell
CRC: Colorectal cancer
CXCL12: C-X-C motif chemokine ligand 12
DC: Dendritic cell
DOX: Doxorubicin
ECM: Extracellular matrix
EPR: Enhanced permeability and retention
FAP-α: Fibroblast activation protein alpha
FDA: Food and Drug Administration
GI: Gastrointestinal
HSA: Human serum albumin
Hb: Hemoglobin
IBR: Ibrutinib
ICD: Immunogenic cell death
IL: Interleukin
INF-γ: Interferon gamma
LCP: PEGylated cationic lipid corona
LPD: LPH: Liposome-protamine-hyaluronic acid
LOXL2: Lysyl Oxidase Like 2
MDSC: Myeloid-derived suppressor cells
p-GEM: Monophosphorylated Gemcitabine
NIR: Near infrared
PC: Pancreatic cancer
PD-1: Programmed cell death protein 1
PDA: Polydopamine
PDT: Photodynamic therapy
PFC: Perfluorocarbon
PLANT: Photosynthetic leaf-inspired abiotic/biotic nano-thylakoid
PLGA: Poly (lactic-co-glycolic acid)
PSC: Pancreatic stellate cell
pSmad2: Phospo-Smad2
PTT: Photothermal therapy
RBC: Red blood cell
ROS: Reactive oxygen species
RT: Radiotherapy
SDT: Sonodynamic therapy
sTRAIL: Secretable form of TNF-related apoptosis-inducing ligand
TAM: Tumor-associated macrophage
TEM: Transmission electron microscope
TGF-β: Transforming growth factor beta
TME: Tumor microenvironment
TLR: Toll-like receptor
T-MP: Tumor-derived antigenic microparticle
TNF-α: Tumour necrosis factor alpha
T-RKP: AE105 modified PEG-pOrn15-pLys10-PTX4
VDA: Vasculature disrupting agents
VEGF: Vascular endothelial growth factor
